# Discoid lupus erythematosus and its progression to systemic lupus erythematosus across age groups: a systematic review

**DOI:** 10.25122/jml-2025-0141

**Published:** 2025-09

**Authors:** Abdulelah Alharbi, Oudai Alamri, Abdulaziz Afandi, Abrar Arbaeen, Ammar Mirza, Abdullah Alahmari, Saif Alshomrani, Marwan Qashqari, Mohammed Alahmadi, Saif Alharthy, Amal Kokandi

**Affiliations:** 1 College of Medicine, King Abdulaziz University, Jeddah, Saudi Arabia; 2 Faculty of Medicine and Health, University of Sydney, Sydney, Australia; 3 College of Medicine, Taibah University, Medina, Saudi Arabia; 4 Department of Medical Laboratory Sciences, Faculty of Applied Medical Sciences, King Abdulaziz University, Jeddah, Saudi Arabia; 5 Department of Dermatology, King Abdulaziz University Hospital, Jeddah, Saudi Arabia

**Keywords:** Discoid lupus erythematosus, DLE, systemic lupus erythematosus, SLE, pediatrics, adults, cutaneous lupus erythematosus

## Abstract

Discoid lupus erythematosus (DLE) is a chronic cutaneous form of lupus characterized by erythematous lesions, dyspigmentation, and scarring that may progress to systemic lupus erythematosus (SLE). This systematic review analyzed epidemiology, clinical patterns, immunologic features, progression rates, and treatment outcomes in 2,814 patients across 72 studies, including 626 pediatric/neonatal and 2,188 adult cases. Female participants predominated in both groups (68.5% in pediatrics; 74.2% in adults), with a higher prevalence among African/African American patients (29.6% in pediatrics and 33.8% in adults). The mean age at diagnosis was 11 years in children and 34 years in adults. Localized lesions were most common in pediatric patients (61.3%) and adult patients (58.7%). Progression to SLE occurred in 30.0% of pediatric cases and 25.4% of adults. Identified risk factors included early-onset disease (in children, <10 years; in adults, <20 years), ANA positivity (51% in pediatric; 48% in adult), high ANA titers (≥1:320), and a family history of rheumatic disease. Treatment relied mainly on topical corticosteroids (44.4% pediatric; 51.6% adult) and hydroxychloroquine (11.1% pediatric; 28.7% adult), while newer therapies such as lenalidomide and anifrolumab showed potential benefits. Overall, DLE demonstrates a strong female predominance and a substantial likelihood of progression to SLE, particularly in younger patients with autoantibody positivity.

## Introduction

Discoid lupus erythematosus (DLE) is a chronic form of cutaneous lupus erythematosus, typically characterized by erythematous, scaly plaques that may evolve into atrophic scars, dyspigmentation, and follicular plugging [1]. Although primarily limited to the skin, DLE may progress to systemic lupus erythematosus (SLE), a multisystem autoimmune disease, complicating both management and prognosis [2].

The global incidence of cutaneous lupus erythematosus (CLE), including DLE, ranges between 3 and 5 cases per 100,000 annually, with higher rates reported in African and African American populations [3,4]. Pediatric-onset DLE is rare, with an estimated incidence of 0.2–0.5 per 100,000 children per year [5]. In adults, DLE accounts for 15–23% of CLE cases [6], most commonly presenting in early to middle adulthood but also documented in older adults [7]. Ethnic disparities are evident: African descent populations experience more severe disease manifestations and higher rates of progression to SLE compared with Caucasian populations [8,9].

While many patients remain with cutaneous-limited disease, 20–30% progress to SLE depending on age, sex, ethnicity, and serologic profile [10,11]. This evolution carries significant implications for prognosis, particularly due to the risk of renal and neurological involvement in systemic disease. Diagnosis is often challenging due to overlap with other dermatoses (e.g., psoriasis, tinea) and the absence of universally accepted diagnostic criteria for DLE [12]. Management remains complicated by heterogeneity in treatment response, limited randomized controlled trial data, and long-term safety concerns associated with systemic therapies, including antimalarials, immunosuppressants, and biologics [13,14].

Although numerous case reports, case series, and retrospective cohorts describe pediatric and adult DLE, the literature remains fragmented, with variability in diagnostic definitions, outcome reporting, and follow-up duration. Synthesizing this evidence is critical to (i) clarify epidemiological trends across age groups, (ii) better understand risk factors for progression to SLE, and (iii) evaluate therapeutic strategies that optimize disease control and minimize long-term morbidity [15–17]. A systematic review, conducted in accordance with PRISMA guidelines, represents the most rigorous method for integrating data across diverse study designs while minimizing bias [18,19].

This systematic review evaluates the epidemiology, clinical features, immunological findings, risk of progression to SLE, and treatment outcomes of DLE across neonates, children, adolescents, and adults. It also highlights knowledge gaps and emerging therapeutic strategies, providing evidence-based insights for dermatologists, rheumatologists, pediatricians, and other clinicians involved in the multidisciplinary care of lupus patients.

## Material and methods

### Study design and registration

This systematic review was conducted in accordance with the PRISMA 2020 guidelines [10]. A completed PRISMA checklist is provided in the Supplementary Materials Table S1. The protocol was registered prospectively in the PROSPERO database (Registration ID: CRD420251033377).

### Eligibility criteria

We included original studies reporting on patients diagnosed with discoid lupus erythematosus across all age groups (neonates, children, adolescents, adults). Eligible study designs included case reports, case series, retrospective and prospective cohort studies, cross-sectional studies, and randomized/non-randomized controlled studies. Studies were included if they reported at least one of the following: (i) demographic characteristics, (ii) clinical presentation, (iii) laboratory/immunological findings, (iv) treatment, or (v) progression to systemic lupus erythematosus (SLE).

Exclusion criteria were: (i) studies without original patient data (e.g., reviews, editorials), (ii) studies focusing solely on systemic lupus erythematosus without cutaneous involvement, and (iii) studies not available in English.

### Information sources and search strategy

A comprehensive search was conducted in MEDLINE (via PubMed), Web of Science, Scopus, and Cochrane Library from inception to December 31, 2024. Additional searches were performed in regional databases (African Index Medicus, LILACS, IndMED) to ensure geographic diversity, given the underrepresentation of African and Latin American populations in prior reviews. Reference lists of included articles were screened for additional eligible studies.

Example (PubMed): ('Discoid Lupus Erythematosus' OR

'DLE' OR 'Cutaneous Lupus') AND ('Children' OR 'Adolescent' OR 'Adult' OR 'Neonate' OR 'Pediatric' OR 'Elderly').

### Study selection

**Figure 1:**
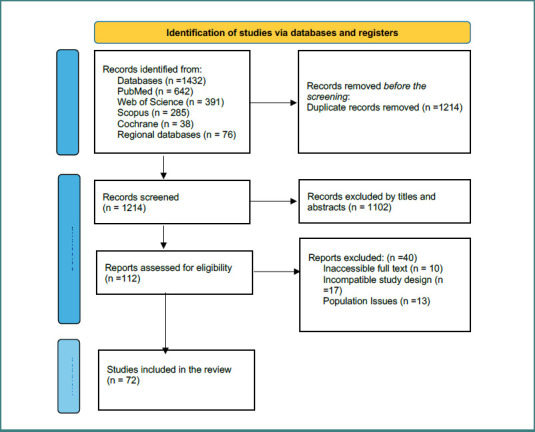
PRISMA flow diagram of study selection process

Titles and abstracts were independently screened by two reviewers. Full-text articles were retrieved when eligibility was unclear. Disagreements were resolved through discussion or consultation with a third reviewer. Duplicates were removed using EndNote X9. The selection process is summarized in Figure 1 (PRISMA flow diagram).

### Data extraction

A standardized extraction form was developed. The following data were extracted:

- Study ID (author, year, country)
- Study design and setting
- Number of participants
- Age, sex, and ethnicity distribution
- Socioeconomic and geographic context (when reported)
- Clinical presentation (lesion type, distribution, systemic features)
- Laboratory/immunologic findings (antinuclear antibodies [ANA], anti-double-stranded DNA antibodies [anti-dsDNA], anti-Smith antibodies [anti-Sm], complement levels, erythrocyte sedimentation rate [ESR])
- Treatments and outcomes
- Progression to SLE (with time to progression when available).

Data were extracted independently by two reviewers and cross-checked for accuracy.

### Risk of bias assessment

Risk of bias was assessed using validated tools tailored to the study design:

- Joanna Briggs Institute (JBI) checklists for case reports (8-item) and case series (10-item) [6,7].
- Newcastle-Ottawa Scale (NOS) for cohort studies (9-star system) [8].
- AXIS tool for cross-sectional studies (20-item) [9].

Studies were categorized as low, moderate, or high risk of bias based on pre-specified cut-offs. Two reviewers independently assessed study quality, with disagreements resolved by consensus.

### Data synthesis and analysis

Due to marked heterogeneity in study design, patient populations, outcome definitions, and follow-up duration, a meta-analysis was not feasible. Instead, we performed a narrative synthesis with descriptive statistics. Proportions are reported with denominators and expressed as percentages with 95% confidence intervals (CI) where data permitted. Subgroup analyses were conducted for pediatric vs. adult populations, and by geographic region and ethnicity when available.

## Results

### Study selection

The initial search identified 1,432 records (PubMed = 642, Web of Science = 391, Scopus = 285, Cochrane = 38, regional databases = 76). After removing 218 duplicates, 1,214 records were screened by title/abstract. A total of 112 full-text articles were reviewed, of which 72 studies met the inclusion criteria. The PRISMA flow diagram is shown in Figure 1.

### Study characteristics

The 72 included studies comprised 39 case reports, 11 case series, 15 retrospective cohort or cross-sectional studies, and 7 prospective or interventional studies, published between 1961 and 2024. Collectively, they reported on 2,814 patients, including 626 pediatric/neonatal and 2,188 adult cases. Geographic distribution was broad, with contributions from North America (34 studies), Europe (19), Asia (12), South America (4), and Africa (3). Across these studies, discoid lupus erythematosus showed a clear female predominance and higher prevalence among African/African American patients. Localized lesions, particularly on the face and scalp, were most frequently reported. Progression to SLE occurred in approximately 30% of pediatric patients and 25% of adult patients. The most common treatments were topical corticosteroids and antimalarials, particularly hydroxychloroquine. These findings are summarized in Table 1, while a detailed matrix of the included studies is provided in the Supplementary Material (Table S2).

**Table 1:** Overview of included studies in the systematic review

Study Design	No. of studies	Total patients (n)	Age groups reported	Key outcomes (progression to SLE, clinical features, treatments)
Case reports	39	58	Neonates, children, adults	Rare presentations of DLE across ages; most localized lesions; treatments included topical corticosteroids, hydroxychloroquine, calcineurin inhibitors; the majority did not progress to SLE.
Case series	11	124	Pediatric & adult	Recurrent patterns of facial/scalp lesions; occasional linear/Blaschkoid variants; some progression to SLE (5–15%); hydroxychloroquine commonly used.
Retrospective cohort / Cross-sectional studies	15	2,412	Children, adolescents, adults	Large cohorts showed female predominance (≈70%), higher prevalence in African/African American patients; progression to SLE ranged 20–30%; ANA positivity frequent; systemic therapies used in disseminated cases.
Prospective/interventional studies	7	220	Adults	Evaluated antimalarials, systemic corticosteroids, and biologics (anifrolumab, lenalidomide); promising short-term outcomes but limited long-term data.

### Demographic data

Among pediatric patients (*n* = 626), the mean age at diagnosis was 11 years (SD ±3.2), with a female predominance of 68.5% (430/626, 95% CI, 64.8–72.0). In adults (*n* = 2,188), the mean age at diagnosis was 34 years (SD ±9.1), with 74.2% females (1,623/2,188, 95% CI: 72.4–76.0). Ethnicity distributions are summarized in Table 2, showing a higher representation of African/African American patients in both groups (29.6% pediatric; 33.8% adult). Socioeconomic and rural/urban classification were inconsistently reported (12 studies).

**Table 2:** Demographic characteristics of patients with discoid lupus erythematosus (DLE)

Variable	Pediatric/Neonatal (*n* = 626)	Adults (*n* = 2,188)	Total (*n* = 2,814)
**Sex**			
Male	196 (31.5%, 95% CI, 27.9–35.2)	565 (25.8%, 95% CI, 24.0–27.6)	761 (27.0%)
Female	430 (68.5%, 95% CI, 64.8–72.0)	1,623 (74.2%, 95% CI, 72.4–76.0)	2,053 (73.0%)
Mean age at diagnosis	11 years (SD ±3.2)	34 years (SD ±9.1)	—
**Ethnicity**			
African / African American	185 (29.6%)	739 (33.8%)	924 (32.8%)
Asian	59 (9.4%)	198 (9.1%)	257 (9.1%)
White / Caucasian	109 (17.4%)	481 (22.0%)	590 (21.0%)
Hispanic / Latino	135 (21.6%)	382 (17.5%)	517 (18.4%)
Middle Eastern / North African (MENA)	17 (2.7%)	74 (3.4%)	91 (3.2%)
Mixed / Multiracial	5 (0.8%)	24 (1.1%)	29 (1.0%)
Not reported	116 (18.5%)	290 (13.2%)	406 (14.4%)
**Geographic region of study**			
North America	210 (33.5%)	1,004 (45.9%)	1,214 (43.1%)
Europe	168 (26.8%)	481 (22.0%)	649 (23.1%)
Asia	143 (22.8%)	352 (16.1%)	495 (17.6%)
South America	62 (9.9%)	154 (7.0%)	216 (7.7%)
Africa	43 (6.9%)	125 (5.7%)	168 (6.0%)

### Clinical presentation

Localized skin lesions predominated in both groups: pediatric (61.3% [384/626]) and adult (58.7% [1,285/2,188]). Facial lesions were most frequent (pediatric: 12.4% [76/626], adult: 14.1% [309/2,188]). Photosensitivity was observed in 23.9% (150/626) of pediatric and 27.8% (607/2,188) of adult patients. Oral/nasal ulcers occurred in 13.5% (85/626) of pediatric cases vs. 16.9% (370/2,188) in adults (Table 3).

**Table 3:** Clinical and laboratory features of patients with discoid lupus erythematosus (DLE)

Feature	Pediatric/Neonatal (*n* = 626)	Adults (*n* = 2,188)	Total (*n* = 2,814)
**Clinical presentation**			
Localized skin lesions	384 (61.3%)	1,285 (58.7%)	1,669 (59.3%)
Disseminated skin lesions	138 (22.0%)	492 (22.5%)	630 (22.4%)
Facial lesions	76 (12.4%)	309 (14.1%)	385 (13.7%)
Scalp lesions/alopecia	42 (6.7%)	198 (9.1%)	240 (8.5%)
Photosensitivity	150 (23.9%)	607 (27.8%)	757 (26.9%)
Oral/nasal ulcers	85 (13.5%)	370 (16.9%)	455 (16.2%)
Other mucocutaneous involvement (e.g., periorbital, ear)	48 (7.7%)	172 (7.9%)	220 (7.8%)
**Laboratory / Immunologic findings**			
ANA positivity	322 (51.4%)	1,052 (48.1%)	1,374 (48.8%)
Anti-dsDNA positivity	137 (21.9%)	427 (19.5%)	564 (20.0%)
Anti-Sm positivity	62 (9.9%)	196 (9.0%)	258 (9.2%)
Hypocomplementemia (low C3/C4)	235 (37.5%)	749 (34.2%)	984 (35.0%)
Elevated ESR/CRP	148 (23.6%)	495 (22.6%)	643 (22.9%)
Hematologic abnormalities (cytopenias)	58 (9.3%)	212 (9.7%)	270 (9.6%)

### Immunological profile

ANA positivity was found in 51.4% (322/626) of pediatric and 48.1% (1,052/2,188) of adult patients. Anti-dsDNA antibodies were present in 21.9% (137/626) of pediatric and 19.5% (427/2,188) of adult patients. Low complement (C3/C4) levels were reported in 37.5% (235/626) of pediatric and 34.2% (749/2,188) of adult patients (Table 3).

### Progression to systemic lupus erythematosus (SLE)

Progression occurred in 188/626 pediatric patients (30.0%, 95% CI, 26.5–33.8%) and 556/2,188 adult patients (25.4%, 95% CI, 23.5–27.5%). Risk factors included early-onset DLE (<10 years in pediatrics; <20 years in adults), ANA positivity with high titers, disseminated lesions, and positive family history of autoimmune disease.

### Treatment patterns

Treatment data were available for 648 patients across case reports and case series (45 pediatric, 603 adult). Topical corticosteroids were the most frequently used therapy in both pediatric (20/45, 44.4%) and adult (311/603, 51.6%) populations. Hydroxychloroquine was the second most common (pediatric: 5/45, 11.1%; adult: 173/603, 28.7%). Other agents included oral corticosteroids, chloroquine derivatives, dapsone, tacrolimus, and newer biologics (e.g., anifrolumab, lenalidomide) in adults (Table 4).

**Table 4:** Treatment patterns in patients with discoid lupus erythematosus (DLE)

Treatment	Pediatric/Neonatal (*n* = 45 with treatment data)	Adults (*n* = 603 with treatment data)	Total (*n* = 648)
Topical corticosteroids	20 (44.4%)	311 (51.6%)	331 (51.1%)
Topical calcineurin inhibitors (tacrolimus/pimecrolimus)	4 (8.9%)	41 (6.8%)	45 (6.9%)
Systemic corticosteroids	6 (13.3%)	159 (26.4%)	165 (25.5%)
Hydroxychloroquine	5 (11.1%)	173 (28.7%)	178 (27.5%)
Chloroquine	3 (6.7%)	48 (8.0%)	51 (7.9%)
Methotrexate / Azathioprine / Mycophenolate	2 (4.4%)	37 (6.1%)	39 (6.0%)
Dapsone	1 (2.2%)	19 (3.1%)	20 (3.1%)
Biologics (e.g., anifrolumab, belimumab, lenalidomide)	0 (0.0%)	26 (4.3%)	26 (4.0%)
Other topical therapies (retinoids, photoprotection)	4 (8.9%)	26 (4.3%)	30 (4.6%)

### Risk of bias assessment

Of the 72 included studies, 38 (52.7%) were judged to be at low risk of bias, 28 (38.9%) at moderate risk, and 6 (8.4%) at high risk. Detailed risk of bias assessments by tool are summarized in Table 5.

**Table 5:** Summary of risk of bias assessment for included studies (*n* = 72)

Risk of bias category	Number of studies	Percentage of included studies
Low risk	38	52.7%
Moderate risk	28	38.9%
High risk	6	8.4%
Total	72	100%

## Discussion

Our review identified 72 papers (39 case reports, 11 case series, 15 retrospective cohort/cross-sectional studies, and 7 prospective/interventional studies) that focus on pediatric, neonatal, and adult DLE. This collective body of literature provides important insights into the epidemiological features, clinical manifestations, and treatment approaches for this complex condition across all age groups.

The analysis revealed a predominance of female patients, comprising 73% of the overall population (68.5% of 626 pediatric cases and 74.2% of 2,188 adult cases). This is consistent with the well-documented female predilection observed in adult-onset DLE as well as SLE [11,12].

The most common ethnicity was African/African American, comprising 29.6% of pediatric and 33.8% of adult cases. This is noteworthy, as the African/African American population is known to experience more complications and more severe manifestations of DLE compared to other ethnic groups. These complications include a higher incidence of dyspigmentation, scarring, and alopecia, which highlights the importance of recognizing individual differences among patients, including racial and ethnic differences, socioeconomic disparities, and geographic representation, and tailoring the management and treatment of each patient accordingly [13].

DLE can affect many different areas of the body, but the most common locations are on the face. The forehead, nose, and cheeks are frequently involved, as these sun-exposed areas tend to be more susceptible to the characteristic discoid lesions of DLE. In addition, the scalp is also a common site of involvement, which can lead to scarring alopecia if not properly managed [13,14].

The progression from DLE to SLE was a key objective of the study. We found that 30% of pediatric (*n* = 188/626, 95% CI, 26.5–33.8%) and 25.4% of adult cases (*n* = 556/2,188, 95% CI, 23.5–27.5%) had progressed from DLE to SLE. A short systematic review focused on the pediatric population reported a lower progression rate of 12%. The only statistically significant risk factor identified was the onset of DLE before the age of 10 years [15]. Conversely, another systematic review that examined cutaneous lupus erythematosus more broadly indicated that the progression to SLE can range from 0% to 31%, with a higher age at diagnosis being a risk factor. Other risk factors included positive ANA, anti-dsDNA, a higher ANA titer (≥1:320), and a positive family history of rheumatic diseases [16].

In another study conducted by Chong *et al*., which examined both pediatric and adult populations, it was reported that the transformation rate was 28%. The identified risk factors included lesions located below the neck, arthralgias or arthritis, photosensitivity, nephropathy, and, as noted in a previous study, positive ANA tests, especially those with high titers [17].

For the treatment of DLE, the most commonly used options include topical steroids (pediatric: 44.4%, 20/45; adult: 51.6%, 311/603) and hydroxychloroquine (pediatric: 11.1%, 5/45; adult: 28.7%, 173/603). These findings correspond with current evidence and guidelines. The first-line treatment for DLE typically involves high-potency topical corticosteroids, such as fluocinonide. Other options include topical calcineurin inhibitors such as tacrolimus, although evidence supporting their effectiveness is limited [18,19]. For the systematic treatment, the first line is hydroxychloroquine, which has strong evidence supporting its efficacy. It also has a relatively favorable side effect profile compared to other systemic therapies [20]. In refractory cases, alternative systemic therapies can be considered, including retinoids, thalidomide, and immunosuppressants such as cyclosporine, azathioprine, and methotrexate. These options carry a higher risk of adverse events [21,22].

A new emerging treatment option includes lenalidomide and anifrolumab, which have shown promising results in recent case reports. These medications are particularly used for DLE cases and open up new avenues for additional treatment options. However, further clinical trials are needed to establish their efficacy and safety [23,24].

Overall, our findings demonstrate that DLE is a cross-age disorder with systemic implications. Clinicians should maintain vigilance for systemic progression in high-risk patients—particularly children, individuals of African descent, and those with disseminated or ANA-positive disease—and adopt an interdisciplinary approach involving dermatologists and rheumatologists. The lack of socioeconomic and regional data in many studies highlights the need for more inclusive and globally representative research [25–30].

## Conclusion

This systematic review demonstrates that discoid lupus erythematosus is a chronic cutaneous condition with a marked female predominance, notable ethnic disparities, and a considerable risk of progression to systemic lupus erythematosus, particularly among younger patients and those with positive autoantibodies. Although topical corticosteroids and antimalarials remain the cornerstone of management, emerging biologic and immunomodulatory therapies show promise for refractory cases. Vigilant long-term monitoring is warranted, especially in high-risk populations, to ensure early detection of systemic progression. Future studies should prioritize the use of standardized diagnostic criteria, prospective longitudinal follow-up, and increased representation of understudied populations to refine risk stratification and optimize treatment strategies across different age groups.

## Supplementary Material



## Data Availability

Further data is available from the corresponding author upon reasonable request. Supplementary materials include the PRISMA checklist, detailed search strategies, and a full study matrix (Supplementary Table S2).
