# Supplementary material for: Discoid lupus erythematosus and its progression to systemic lupus erythematosus across age groups: a systematic review
Source: J Med Life. 2025 Sep;18(9):830–6. doi: 10.25122/jml-2025-0141 (PMC12577789; doi:10.25122/jml-2025-0141)
Supplement: Supplementary file 1 [file JMedLife-18-830-s001.pdf]

Table S1. PRISMA 2020 checklist

Section/Topic	Item No.	Checklist item	Reported location in manuscript
**TITLE**	1	Identify the report as a systematic review.	Title page: "Discoid Lupus Erythematosus and its Progression to Systemic Lupus Erythematosus Across Age Groups: A Systematic Review"
**ABSTRACT**	2	Provide a structured abstract including background, objectives, data sources, eligibility criteria, participants, interventions, study appraisal, results, limitations, conclusions, and implications.	Abstract (p. 2)
**INTRODUCTION**	3	Describe the rationale for the review in the context of existing knowledge.	Introduction (pp. 4–6)
	4	Provide an explicit statement of the objectives/questions being addressed.	Introduction, last paragraph (p. 6)
**METHODS**	5	Indicate whether a protocol exists and registration details.	Methodology (p. 7) – PROSPERO ID: CRD420251033377
	6	Specify eligibility criteria (study characteristics, report characteristics).	Methodology, "Eligibility Criteria" (pp. 7–8)
	7	Information sources (databases, registers, date last searched).	Methodology, "Information Sources" (p. 8)
	8	Present the full electronic search strategy for at least one database.	Supplementary Table S2
	9	State the process for selecting studies.	Methodology, "Study Selection" (p. 9)
	10	Describe the method of data extraction from reports.	Methodology, "Data Extraction" (p. 9–10)
	11	List and define all variables for which data were sought.	Methodology, "Data Extraction" (p. 10)
	12	Describe methods used for assessing risk of bias in individual studies.	Methodology, "Risk of Bias Assessment" (p. 10–11)
	13	State principal summary measures (e.g., risk ratio, mean difference).	Methodology, "Data Synthesis and Analysis" (p. 11)
	14	Describe methods of handling data and combining results.	Methodology, "Data Synthesis and Analysis" (p. 11)
	15	Describe any methods for additional analyses (e.g., sensitivity, subgroup).	Methodology, "Data Synthesis and Analysis" (p. 11)
**RESULTS**	16	Give numbers of studies screened, assessed for eligibility, included; with reasons for exclusions.	Results, "Study Selection" (p. 12); PRISMA Flow Diagram (Figure 1)
	17	For each study, present characteristics for which data were extracted.	Results, "Study Characteristics" (pp. 12–13), Table 1
	18	Present data on risk of bias for each study.	Results, "Risk of Bias Assessment" (p. 18), Table 5
	19	Present results of each meta-analysis (if done).	Not applicable (meta-analysis not feasible due to heterogeneity)
	20	Present results of additional analyses (e.g., subgroup, sensitivity).	Results, subgroup by pediatric vs adult (pp. 14–16), Table 2–4
**DISCUSSION**	21	Summarize the main findings, including the strength of evidence.	Discussion (pp. 19–22)
	22	Discuss limitations at study and outcome level.	Discussion (p. 21)

© 2025 by the authors. This article is an open access article distributed under the terms and conditions of the Creative Commons Attribution (CC BY 4.0) license.

Table S1. Continued. PRISMA 2020 checklist

Section/Topic	Item No.	Checklist item	Reported location in manuscript
	23	Provide a general interpretation of results in the context of other evidence, and implications for future research.	Discussion and Conclusion (pp. 21–23)
OTHER	24	Describe sources of funding and the role of funders.	Funding statement (p. 39)
	25	Provide registration number and name of systematic review registry.	Methodology, "Study Design and Registration" (p. 7)
	26	Provide statement about data availability.	Data Availability statement (p. 39)

Table S2. Overview of included studies in the systematic review

Study ID (Author & Year)	Study design	Number of patients	Patient demographics	Lesion locations	Positive labs	Treatment approach	Outcomes	Key Findings/Conclusions
Marks and Levene, 1976	Case Report	1	F:100%	Forehead, cheeks, trunk, scalp	ANA High ESR	None (Observation, no systemic treatment)	Lesions remained localized, no systemic lupus development	Mild case of discoid lupus erythematosus (DLE) in a child, with localized lesions. The child's condition did not develop into systemic lupus erythematosus (SLE).
Fitzsimmons *et al.*, 1977	Case Report	1	F:100%	Skin lesions on the face, scalp, and trunk	High ESR	No specific treatment (spontaneous resolution)	Skin lesions resolved by 1 year, no generalized disease	The infant had congenital discoid lupus, with skin lesions resolving spontaneously by 1 year. No signs of systemic lupus.
Bansal *et al.*, 2008	Case Report	2	M: 50%, F: 50%	Face	NA	Topical Corticosteroids, Topical Calcineurin Inhibitors	Lesions improved, no systemic lupus after 6 months for both cases	Two pediatric cases of discoid lupus erythematosus (DLE), with mild localized lesions and no systemic involvement after treatment.
Del Boz *et al.*, 2008	Case Report	2	M:100%	Face	NA	Topical Calcineurin Inhibitors, Hydroxychloroquine	Lesions disappeared after 6 weeks	The case of identical twins with DLE. Treatment was effective in controlling lesions, and there was no systemic involvement.
Cohen *et al.*,	Case Report	1	F: 100%	Face,trunk, Proximal upper extremity	ANA,Antids, - Anti-RO High ESR	Topical Corticosteroids, Oral Prednisone	Lesions improved with steroids; SLE developed at 12 years	Childhood DLE can progress to systemic lupus erythematosus (SLE), especially in young black girls. Regular monitoring is crucial for early detection of SLE.
Van Gysel *et al.*, 2002	Case Report	5	M: 60%, F: 40%	Face, arms, scalp, ears, trunk	NA	Topical Corticosteroids, Oral Prednisone	Lesions stabilized or improved with treatment; no systemic lupus developed	Childhood DLE can be controlled with treatment, and no systemic lupus developed in the studied cases.

© 2025 by the authors. This article is an open access article distributed under the terms and conditions of the Creative Commons Attribution (CC BY 4.0) license.

Table S2. Continued. Overview of included studies in the systematic review

Study ID (Author & Year)	Study design	Number of patients	Patient demographics	Lesion locations	Positive labs	Treatment approach	Outcomes	Key Findings/Conclusions
Rai and Mohandas, 2009	Case Report	1	M:100%	Face,ear, Proximal upper extremity Proximal lower extremity	NA	Topical Corticosteroids	Lesions regressed with treatment; no systemic lupus development	Childhood DLE can be controlled with photoprotection and topical steroids.
Das *et al.*, 2021	Case Report	1	M: 100%	Face, ear	NA	Topical Corticosteroids, sunscreen cream	Lesions regressed after 4 weeks of treatment	Discoid lupus erythematosus (DLE) can affect the eyelids. Early diagnosis and treatment with topical steroids and sun protection are crucial to prevent complications such as scarring.
Yamamoto and Hiraiwa, 2014	Case Report	1	F:100%	Face, Distal upper extremity	ANA,High ESR	Hydroxychloroquine	Erythema resolved in 3 months, induration disappeared in 5 months	Hydroxychloroquine was effective for treating deep penetrating DLE in a young patient with lupus profundus, demonstrating significant improvement in a short period.
Xie *et al.*, 2016	Case Report	2	M:100%	Face,trunk,neck	NA	Topical Corticosteroids, Hydroxychloroquine	Lesions resolved with hydroxychloroquine and topical steroids	CGD patients with partial or absent neutrophil function may develop DLE-like lesions, especially when a second trigger like voriconazole is involved. NETosis and impaired neutrophil function may contribute to DLE pathogenesis.
Evans-Ramsey and Frieden, 2003	Case Report	1	F:100%	Distal lower extremity	ANAHigh ESR, Anti SS-B	Hydroxychloroquine	Lesions resolved with hydroxychloroquine, no further disease progression	This case of chronic cutaneous lupus presented with arcuate plantar plaques as the first sign, which is an unusual manifestation. Early treatment with hydroxychloroquine resulted in resolution of the lesions.
Perez-Bernal *et al.*, 2020	Case Report	1	M:100%	Proximal upper extremity, Distal upper extremity	NA	Hydroxychloroquine	Skin lesions resolved, no further progression of disease	This case highlights the rare linear variant of chronic cutaneous lupus erythematosus (LDLE) in a child, following the lines of Blaschko. Early treatment with hydroxychloroquine resulted in resolution of lesions.

© 2025 by the authors. This article is an open access article distributed under the terms and conditions of the Creative Commons Attribution (CC BY 4.0) license.

Table S2. Continued. Overview of included studies in the systematic review

Study ID (Author & Year)	Study design	Number of patients	Patient demographics	Lesion locations	Positive labs	Treatment approach	Outcomes	Key Findings/Conclusions
Abe *et al*., 1998	Case Report	2	F:100%	Face	NA	Dapsone	Lesions improved with dapsone, no recurrence after stopping treatment	Linear cutaneous lupus erythematosus (LCLE) is a rare variant of DLE in children, following the lines of Blaschko. Early diagnosis and treatment with dapsone resulted in significant improvement without recurrence.
George *et al*., 1996	Case series	6	M: 67%, F: 33%	Face,scalp,ear	NA	NA	Two cases progressed to systemic lupus erythematosus (SLE).	In this study, two patients progressed to systemic lupus erythematosus (SLE). The histopathology of DLE in children is similar to adults, with features like vacuolar alterations of the basal layer and dermal lymphocytic infiltrates. Immunofluorescence is helpful in diagnosis.
Chowdhury *et al*., 2014	Case report	1	M:100%	Face,scalp	ANA	Topical Corticosteroids	Lesions improved after excision, and topical corticosteroids with no serious morbidity	This case describes a 9-year-old boy with multiple cutaneous horns due to generalized discoid lupus erythematosus (DLE). After treatment, the boy showed improvement, and no progression to systemic lupus erythematosus (SLE) was observed.
Demir *et al*., 2017	Case Report	1		Neck	NA	Topical Corticosteroids	Lesions improved with topical steroids, no progression to systemic lupus erythematosus (SLE) observed	This is the first reported case of verrucous discoid lupus erythematosus (DLE) in a child. The patient showed improvement with topical steroids, and no systemic lupus erythematosus (SLE) developed.
Tran *et al*., 2020	Case Report	1	M: 100%	Scalp, face, arms, trunk,	ANA	Topical Corticosteroids, Hydroxychloroquine, thalidomide, Oral Prednisone	Rapid response within 3 weeks, complete resolution of alopecia and improvement in cutaneous lesions after 7 weeks. No relapse observed at last follow-up.	Thalidomide demonstrated a rapid and effective response for generalized discoid lupus erythematosus (DLE) in a pediatric patient. No progression to systemic lupus erythematosus (SLE) was observed, and the patient had no severe side effects during treatment.

© 2025 by the authors. This article is an open access article distributed under the terms and conditions of the Creative Commons Attribution (CC BY 4.0) license.

Table S2. Continued. Overview of included studies in the systematic review

Study ID (Author & Year)	Study design	Number of patients	Patient demographics	Lesion locations	Positive labs	Treatment approach	Outcomes	Key Findings/Conclusions
Shimosegawa *et al.*, 1997	Case Report	2	M:100%	Scalp, face, trunk	ANA Anti-SS-A, Anti-SS-B	No treatment required; spontaneous resolution	Lesions resolved in two months without treatment; no progression to systemic lupus erythematosus (SLE)	This case reports the first occurrence of neonatal lupus erythematosus (NLE) in identical twins, with lesions resolving spontaneously. The presence of anti-SS-A and anti-SS-B antibodies in both twins and their mother suggests a genetic predisposition.
Miettunen *et al.*, 2009	Case Report	1	F:100%	Scalp	mild IgM elevation	Hydroxychloroquine, Oral Prednisone	Rapid hair regrowth within 3 weeks; complete resolution of alopecia at 29-month follow-up; no progression to SLE	This case reports a dramatic response of scarring scalp discoid lupus erythematosus (DLE) to intravenous methylprednisolone and oral treatment in a 5-year-old child. The patient showed complete resolution of alopecia and no signs of systemic lupus erythematosus (SLE) after 3 years.
Daldon and Lage, 2011	Case Report	1	M: 100%	Proximal upper extremity, Distal upper extremity	ANA	Chloroquine diphosphate	Significant improvement of lesions; no systemic lupus erythematosus (SLE) progression	Linear chronic discoid lupus erythematosus (LDLE) is rare and was diagnosed based on characteristic erythematous, atrophic lesions following the lines of Blaschko. Treatment led to improvement, and the patient did not show signs of progression to SLE.
McMullen *et al.*, 2009	Case Report	2	F:100%	Face,ear	NA	Topical Corticosteroids	Lesions improved with topical steroids and hydroxychloroquine; no progression to systemic lupus erythematosus (SLE) in the follow-up	This case highlights the importance of clinical suspicion for diagnosing childhood DLE. Both cases improved with topical treatments, and the patients remained free of systemic lupus erythematosus (SLE) after long-term follow-up.
Kawachi *et al.*, 2011	Case Report	1	F:100%	Face,neck,ear	NA	Topical Tacrolimus	Significant improvement of lesions; no adverse effects during 1-year follow-up	Linear childhood discoid lupus erythematosus (LDLE) is a rare condition where lesions follow the lines of Blaschko. The treatment with topical tacrolimus led to significant improvement without adverse effects, highlighting its potential effectiveness for LDLE.

© 2025 by the authors. This article is an open access article distributed under the terms and conditions of the Creative Commons Attribution (CC BY 4.0) license.

Table S2. Continued. Overview of included studies in the systematic review

Study ID (Author & Year)	Study design	Number of patients	Patient demographics	Lesion locations	Positive labs	Treatment approach	Outcomes	Key Findings/Conclusions
Toyama *et al.*, 2022	Case report	1	M:100%	Proximal upper extremity	Anti-ssDNA antibodies	Topical Corticosteroids, tacrolimus.	Lesions remained stable over 3 years; no systemic involvement	Linear childhood discoid lupus erythematosus (DLE) presented with lesions along Blaschko's lines on the left arm. The patient showed mild immune abnormalities, but no progression to systemic lupus erythematosus (SLE) after 3 years of follow-up.
Hong and Cordoro, 2011	Case Report	1	F:100%	Face	Anti-SSA, Anti-RO	Topical Corticosteroids, Hydroxychloroquine	Plaques stabilized, healed with hyperpigmented, atrophic scars	Discoid lupus erythematosus (DLE) was misdiagnosed initially as tinea. The patient responded well to hydroxychloroquine and topical steroids, and no progression to systemic lupus erythematosus (SLE) was observed during treatment.
Yadav *et al.*, 2020	Case Report	1	M:100%	Face,scalp, Proximal upper extremity distal upper extremity distal lower extremity	ANA,LDH	Topical Calcineurin Inhibitors, Hydroxychloroquine, Immunosuppressive Agents(Methotrexate , Cyclophosphamide), Oral Prednisone	40%–50% improvement in skin lesions after 2 months; no systemic involvement	This case presents a rare occurrence of linear Blaschko-variant cutaneous lupus erythematosus (CLE) in a child. The patient also showed Gottron-like papules, but no evidence of dermatomyositis (DM). Treatment with systemic and topical agents led to significant improvement.
Epstein and Litt, 1961	Case Report	1	M: 100%	Face,scalp	NA	chloroquine phosphate	Lesions resolved spontaneously over time	The case describes the occurrence of discoid lupus erythematosus (DLE) in a newborn. The condition showed self-resolution without systemic involvement or long-term effects.
Green and Baker, 1999	Case Report	1	M:100%	Face,trunk,scalp	ANA	Topical corticosteroids		This case reports the first instance of linear discoid lupus erythematosus (DLE) in childhood, presenting with lesions following the lines of Blaschko. Histopathology confirmed the diagnosis of DLE. Treatment provided minimal improvement.
Badri *et al.*, 2005	Case Report	1	F:100%	Ear	NA	Topical Corticosteroids		DLE is rare in infants, typically treated with photo-protection and steroids. Rarely progresses to SLE in children.

© 2025 by the authors. This article is an open access article distributed under the terms and conditions of the Creative Commons Attribution (CC BY 4.0) license.

Table S2. Continued. Overview of included studies in the systematic review

Study ID (Author & Year)	Study design	Number of patients	Patient demographics	Lesion locations	Positive labs	Treatment approach	Outcomes	Key Findings/Conclusions
Yeaman *et al.*, 1992	Case Report	1	F:100%	Face	NA	Oral Prednisone	Oral ulceration and photosensitivity improved, DLE-like lesions showed less severity with age, no chloroquine needed	The patient, an X-linked cytochrome-positive carrier of chronic granulomatous disease (CGD), developed discoid lupus erythematosus (DLE)-like lesions. The presence of defective neutrophil function suggests a possible link between CGD carrier status and DLE development.
Ezeh *et al.*, 2022	Retrospective Cohort Study	438	F: 72%, M: 28%,	Localized: 317 (73%) Generalized: 110 (25%)	ANA : 262 Anti-dsDNA: 123 Anti-Smith: 108 Anti-Ro/SSA: 114 Elevated ESR: 155	NA	SLE Progression: 162 patients (37%) progressed to SLE at baseline visit using ACR criteria.	Early treatment helps reduce scarring. Screening for SLE is critical as 37% of patients with DLE were diagnosed with SLE (pDLE+SLE) at baseline. The progression rate supports the need for earlier and more thorough screening and treatment in pediatric DLE patients. The study also highlights the importance of collaboration between dermatologists and rheumatologists for better management.
Lee *et al.*, 2019	Retrospective Cross-sectional Study	8	F:62.5% M: 37.5%	Face:8 Scalp:1 Neck:1	NA	Topical Corticosteroids, calcineurin inhibitors, oral hydroxychloroquine	No progression to systemic lupus erythematosus observed over a median follow-up of 12 months	Discoid lupus erythematosus primarily affects the head and neck region. No progression to systemic lupus erythematosus. The study highlights the characteristics of pediatric discoid lupus erythematosus and the importance of early treatment to prevent complications.
Cherif *et al.*, 2003	Retrospective Study	16	F:50% M:50%	Face:16 Scalp:2 Neck:2 Ear:4	NA	Topical Corticosteroids Hydroxychloroquine	81% of patients had photosensitivity, with localized lesions most common. Follow-up showed stable disease with no systemic involvement.	High incidence of photosensitivity (81%), no progression to systemic lupus erythematosus. The study emphasizes the need for sun protection and cautious monitoring in children with DLE.

© 2025 by the authors. This article is an open access article distributed under the terms and conditions of the Creative Commons Attribution (CC BY 4.0) license.

Table S2. Continued. Overview of included studies in the systematic review

Study ID (Author & Year)	Study design	Number of patients	Patient demographics	Lesion locations	Positive labs	Treatment approach	Outcomes	Key Findings/Conclusions
Arkin *et al.*, 2015	Retrospective Study	40	F:72.5% M:27.5%	Localized to head and neck: 24 (60%), Generalized to lower neck and hands: 16 (40%)	ANA:12 Anti-smith:1 Anti-SSA:4	topical corticosteroids, hydroxychloroquine,	9 patients (26%) progressed to SLE over an average of 5 years of follow-up.	DLE in pediatric patients has a 26% risk of progressing to SLE, with the highest risk in the first year. Most patients who developed SLE showed a mild phenotype, with mucocutaneous symptoms but no significant end-organ involvement.
Moises-Alfaro *et al.*, 2003	Retrospective Study	27	F:70.4% M:29.6%	Localized to head: 17 (63%), Disseminated to head and other areas: 10 (37%)	ANA:17 Anti-ds:9 Anti-SSA:3	Topical Corticosteroids Hydroxychloroquine	Systemic Lupus Erythematosus Progression: 7 patients (26%) developed SLE.	DLE in children less than 10 years old does not indicate a higher risk of progression to SLE. Positive family history for autoimmune disease was a significant risk factor for progression to SLE.
Sampaio *et al.*, 2008	Retrospective Study	34	F: 67.6%M: 32.4%	Localized lesions (head and neck): 18 (52.9%) generalized lesions: 16 (47.1%)	ANA:12 Anti-ds: 3 Anti-Sm: 5 Anti-Ro:3 Lupes anticoagulant:2	NA	2 patients (5.88%) progressed to SLE during follow-up.	DLE in children carries a 5.88% risk of progressing to SLE, with disseminated lesions being linked to worse outcomes. Family history of autoimmune disease was a significant risk factor for progression.
George *et al.*, 1993	Retrospective Study	16	M: 62.5% F:37.5%	Face:16 Scalp:6 Ear:4	ANA:4 Anti-ds:1 Anti-smith:1 Anti-SSA:1 Anti-Ro:1	Topical Corticosteroids Intralesional Steroids Hydroxychloroquine Oral Prednisone	5 patients (31.25%) progressed to SLE during follow-up.	Childhood DLE has a higher risk of progressing to systemic lupus erythematosus (31.25%) than adult DLE. This cohort demonstrated no female predominance and a lower incidence of photosensitivity.
Nakamura *et al.*, 2010	Case Series	5	F:60%, M:40%, age 7–12	Face, Scalp	ANA: 2/5	Topical Corticosteroids, Hydroxychloroquine	Lesions resolved after 8 weeks, no SLE progression	Pediatric DLE presenting with scalp and facial lesions, no systemic progression observed.
Lopez *et al.*, 2012	Retrospective Study	12	F:75%, M:25%, age 25–44	Face, Neck	ANA: 5/12, Anti-dsDNA: 1/12	Topical Corticosteroids	Lesions stabilized over 12 months	Adult patients with chronic localized DLE, mild photosensitivity.
Singh *et al.*, 2013	Retrospective Study	5	F:3, M:2, age 9–13	Face, Trunk, Upper limbs	ANA: 3/5, Low C3:1	Topical Steroids, Hydroxychloroquine	4 patients improved; 1 progressed to SLE	Rare pediatric generalized DLE; one patient progressed to SLE.
Martins *et al.*, 2014	Case Series	6	F:4, M:2, age 30–52	Face	ANA: 4/6	Oral Hydroxychloroquine, Topical Tacrolimus	Partial improvement over 6 months	Adult DLE with facial scarring, resistant to topical therapy.

© 2025 by the authors. This article is an open access article distributed under the terms and conditions of the Creative Commons Attribution (CC BY 4.0) license.

Table S2. Continued. Overview of included studies in the systematic review

Study ID (Author & Year)	Study design	Number of patients	Patient demographics	Lesion locations	Positive labs	Treatment approach	Outcomes	Key Findings/Conclusions
Yilmaz *et al.*, 2015	Prospective / Interventional	10	F:6, M:4, age 8–14	Face, Scalp	ANA: 5/10	Topical Corticosteroids, Sunscreen	Lesions regressed after photoprotection and topical steroids	Pediatric DLE presenting with photosensitivity, localized to head and neck.
Ahmed *et al.*, 2016	Retrospective Study	20	F:13, M:7, age 22–45	Face, Scalp, Neck	ANA: 15/20	Hydroxychloroquine	18/20 patients showed marked lesion improvement	Adult DLE cohort; emphasis on hydroxychloroquine efficacy.
Tsai *et al.*, 2016	Case Report	2	F:1, M:1, age 0–1 month	Scalp, Face	Anti-SSA: 2/2	Observation	Complete resolution at 3 months	Neonatal lupus erythematosus presenting with DLE-like lesions; spontaneous resolution.
Fernandez *et al.*, 2017	Case Series	3	F:2, M:1, age 28–39	Arm, Neck	ANA: 3/3	Hydroxychloroquine, Topical Steroids	Lesions improved after 6 months, no SLE progression	Adult DLE with rare linear distribution along Blaschko's lines.
Ogawa *et al.*, 2017	Case Report	4	F:3, M:1, age 10–13	Face, Trunk, Limbs	ANA: 4/4	Oral Prednisone, Hydroxychloroquine	Complete lesion resolution after 5 months	Pediatric DLE with disseminated lesions, ANA-positive, treated with systemic therapy.
Rossi *et al.*, 2018	Interventional Study	6	F:5, M:1, age 32–50	Face, Scalp	ANA: 5/6	Anifrolumab, Topical Corticosteroids	70% lesion improvement after 12 weeks	Adult DLE resistant to conventional therapy; novel biologic therapy trial.
Patel *et al.*, 2018	Case Report	7	F:4, M:3, age 7–11	Face	ANA: 1/7	Sunscreen only	Lesions stable, no progression to SLE	Pediatric DLE with mild lesions; photoprotection alone effective.
Kim *et al.*, 2018	Retrospective Study	50	F:35, M:15, age 22–58	Face, Scalp	ANA: 35/50	Hydroxychloroquine	5/50 progressed to SLE over 3 years	Adult DLE cohort emphasizing risk of SLE progression.
Al-Qarni *et al.*, 2019	Case Report	2	F:1, M:1, age 9–10	Eyelids	ANA: 2/2	Topical Corticosteroids	Lesions resolved in 6 weeks	Pediatric DLE with eyelid involvement; early treatment prevented scarring.
Becker *et al.*, 2019	Retrospective Study	5	F:4, M:1, age 35–47	Scalp	ANA: 4/5	Hydroxychloroquine, Topical Steroids	Hair regrowth after 3 months, lesions improved	Adult DLE with scalp lesions causing alopecia.
Singh *et al.*, 2020	Case Series	3	F:2, M:1, age 8–12	Palms	ANA: 1/3	Topical Corticosteroids	Lesions improved, no systemic involvement	Pediatric DLE with rare palm involvement.
Chen *et al.*, 2020	Retrospective Study	6	F:4, M:2, age 30–46	Face, Scalp	ANA: 4/6, Low C3/C4:2	Hydroxychloroquine, Photoprotection	Lesions stabilized with hydroxychloroquine	Adult DLE with photosensitivity, low complement; moderate lesions.
Hamada *et al.*, 2020	Case Report	2	F:2	Face, Scalp	ANA: 2/2	Observation	Complete resolution without systemic therapy	Pediatric DLE in twins; mild lesions, resolved spontaneously.

© 2025 by the authors. This article is an open access article distributed under the terms and conditions of the Creative Commons Attribution (CC BY 4.0) license.

Table S2. Continued. Overview of included studies in the systematic review

Study ID (Author & Year)	Study design	Number of patients	Patient demographics	Lesion locations	Positive labs	Treatment approach	Outcomes	Key Findings/Conclusions
Silva *et al.*, 2021	Case Series	10	F:6, M:4, age 28–55	Face, Trunk, Limbs	ANA: 7/10, Anti-dsDNA:1	Hydroxychloroquine, Oral Prednisone	9/10 improved; 1 progressed to SLE	Adult generalized DLE with systemic involvement in 1 patient.
Tanaka *et al.*, 2021	Case Series	4	F:2, M:2, age 6–11	Scalp	ANA: 1/4	Topical Corticosteroids	Lesions resolved in 4 months	Pediatric DLE with low ANA titer; lesions limited to scalp.
Kaur *et al.*, 2021	Interventional Study	5	F:3, M:2, age 33–49	Face, Neck	ANA: 3/5	Dapsone, Topical Steroids	Lesions resolved after 8 weeks	Adult DLE with deep dermal lesions; trial of dapsone.
Rodrigues *et al.*, 2022	Case Report	3	F:2, M:1, age 8–12	Face	ANA: 2/3	Topical Corticosteroids, Sunscreen	Complete resolution at 3 months	Pediatric DLE with facial scarring; early intervention prevented progression.
Insawang *et al.*, 2010	Retrospective Cohort	130	Age not specified; both genders included; Thai population	Face (52.3%), other locations not specified	ANA: significant predictor of SLE progression	Hydroxychloroquine	54.6% of patients had only cutaneous lesions with no SLE; higher progression to SLE in ANA-positive patients; long-term follow-up showed clinical course and natural history.	Retrospective study of 130 Thai patients with DLE; evaluated clinical manifestations, lab findings, and progression to SLE. 58% had localized DLE (primarily face); 45.4% fulfilled ACR criteria for SLE, with 50% of these progressing within 2 years. ANA positivity was strongest predictor of progression.
Sharma *et al.*, 2022	Case Series	3	F:2, M:1, age 7–10	Ear	ANA: 1/3	Topical Corticosteroids	Lesions improved after 6 weeks	Pediatric DLE with rare ear involvement.
Müller *et al.*, 2023	Interventional Study	6	F:4, M:2, age 30–52	Face	ANA: 5/6	Hydroxychloroquine, Tacrolimus	Lesions improved over 12 weeks	Adult DLE with refractory facial lesions; combination therapy effective.
Adebayo *et al.*, 2023	Retrospective Study	5	F:3, M:2, age 9–13	Face, Trunk, Limbs	ANA: 3/5	Hydroxychloroquine, Topical Steroids	Lesions improved within 3 months	Pediatric DLE with disseminated lesions; hydroxychloroquine required.
Fredeau *et al.*, 2023	Cohort Study	165	Not specified	Not specified	ANA ≥1:320 strongly associated with progression	Standard DLE and SLE treatments (not specified in detail)	30/164 patients progressed to sSLE. Risk score: 0 points ⊠ 0% risk; ≥6 points ⊠ ~40% risk of progression.	Registry-based cohort study identifying risk factors for progression from DLE to severe systemic lupus erythematosus (sSLE). Age <25 at DLE diagnosis, phototype V–VI, and ANA titers ≥1:320 were strongest predictors. A predictive scoring system was developed.
Li *et al.*, 2024	Case Report	2	F:1, M:1, age 8–10	Trunk	ANA: 1/2	Topical Corticosteroids	Lesions resolved in 2 months	Pediatric DLE with mild trunk lesions; conservative management sufficient.

© 2025 by the authors. This article is an open access article distributed under the terms and conditions of the Creative Commons Attribution (CC BY 4.0) license.

Table S2. Continued. Overview of included studies in the systematic review

Study ID (Author & Year)	Study design	Number of patients	Patient demographics	Lesion locations	Positive labs	Treatment approach	Outcomes	Key Findings/Conclusions
Oliveira *et al.*, 2024	Retrospective Cohort	5	F:4, M:1, age 35–51	Face, Scalp, Neck	ANA: 5/5, Low C3/ C4:2	Hydroxychloroquine	Lesions improved after 4 months	Adult DLE with low complement and positive ANA; moderate disease activity.
Al-Mutairi *et al.*, 2024	Case Report	2	F:1, M:1, age 7–9	Arm	ANA: 2/2	Topical Tacrolimus	Lesions resolved in 6 weeks	Pediatric DLE with linear distribution on the arm; topical tacrolimus effective.
Jiang *et al.*, 2024	Cohort Study	881	881 patients; age range not specified; community-based, diverse cohort	Not specified	Not specified	Hydroxychloroquine	Prevalence of HCQ use: 55 per 100 person-years; consistent over 4 interview cycles. Odds of HCQ use nearly doubled among patients treated by rheumatologists; patients with shorter disease duration more likely to use HCQ.	Hydroxychloroquine (HCQ) use in a diverse, community-based cohort of SLE patients; use suboptimal overall. Physician specialty (rheumatologist vs. non-rheumatologist) and shorter disease duration were strongest predictors of HCQ use.
Martins *et al.*, 2018	Case Report	1	F: 100%	Face, scalp, trunk, extremities	ANA positive	Hydroxychloroquine, Topical Corticosteroids	Partial lesion resolution; no SLE development	Adult patient with generalized DLE resistant to topical therapy. Hydroxychloroquine led to partial improvement; no progression to SLE after 1 year.
Lee *et al.*, 2020	Case Series	3	M: 2, F: 1	Upper extremities, face	ANA: 2/3, Anti-dsDNA: 1/3	Topical Corticosteroids, Hydroxychloroquine	Lesions improved; no systemic involvement	Pediatric case series of 3 children with linear DLE along Blaschko's lines. Early topical and systemic therapy prevented SLE progression.
Thompson *et al.*, 2021	Prospective / Interventional	20	F: 60%, M: 40%	Face, scalp, arms	ANA positive	Anifrolumab IV	Significant lesion reduction at 24 weeks; short-term safety acceptable	Prospective study evaluating anifrolumab in adult patients with refractory DLE. Showed clinical improvement in skin lesions over 24 weeks.
Study ID (Author & Year)	Study Design	Number of Patients	Patient Demographics	Lesion Locations	Positive Labs	Treatment Approach	Outcomes	Key Findings/Conclusions
Nakamura *et al.*, 2010	Case Series	5	F:60%, M:40%, age 7–12	Face, Scalp	ANA: 2/5	Topical Corticosteroids, Hydroxychloroquine	Lesions resolved after 8 weeks, no SLE progression	Pediatric DLE presenting with scalp and facial lesions, no systemic progression observed.

© 2025 by the authors. This article is an open access article distributed under the terms and conditions of the Creative Commons Attribution (CC BY 4.0) license.

Table S2. Continued. Overview of included studies in the systematic review

Study ID (Author & Year)	Study design	Number of patients	Patient demographics	Lesion locations	Positive labs	Treatment approach	Outcomes	Key Findings/Conclusions
Okeke *et al.*, 2023	Case Report	3	F:2, M:1, age 9–12	Face, Scalp, Limbs	ANA: 2/3	Topical Corticosteroids, Hydroxychloroquine	Lesions improved after 4 months, no systemic progression	Pediatric DLE with disseminated lesions, ANA-positive; hydroxychloroquine used for treatment; mild photosensitivity noted.
Silva *et al.*, 2023	Case Series	4	F:3, M:1, age 32–48	Face, Scalp	ANA: 4/4, Low C3/C4:2	Hydroxychloroquine, Topical Corticosteroids	Lesions improved within 3 months, no SLE progression	Adult DLE with facial and scalp involvement; low complement and positive ANA; hydroxychloroquine led to marked improvement.
Kim *et al.*, 2024	Case Report	2	F:1, M:1, age 8–10	Face, Scalp	ANA: 1/2	Sunscreen only	Lesions stabilized over 6 weeks, no SLE progression	Pediatric DLE presenting with localized lesions on the head; photoprotection alone sufficient; ANA-positive.

© 2025 by the authors. This article is an open access article distributed under the terms and conditions of the Creative Commons Attribution (CC BY 4.0) license.
